# The Neglected Uterine NK Cells/Hamperl Cells/Endometrial Stromal Granular Cell, or K Cells: A Narrative Review from History through Histology and to Medical Education

**DOI:** 10.3390/ijms241612693

**Published:** 2023-08-11

**Authors:** Lenka Lapides, Ivan Varga, Mária Csöbönyeiová, Martin Klein, Lada Pavlíková, Kristína Visnyaiová, Pavel Babál, Renáta Mikušová

**Affiliations:** 1 Institute of Histology and Embryology, Faculty of Medicine, Comenius University in Bratislava, Sasinkova 4, 811 08 Bratislava, Slovakia; lenka.lapides@gmail.com (L.L.); maria.csobonyeiova@fmed.uniba.sk (M.C.); martin.klein@fmed.uniba.sk (M.K.); renata.mikusova@fmed.uniba.sk (R.M.); 2 Faculty of Health Care Studies, University of Western Bohemia, 30100 Pilsen, Czech Republic; lpavliko@kfe.zcu.cz; 3 Faculty of Medicine, Comenius University in Bratislava, Spitalska 24, 842 15 Bratislava, Slovakia; visnyaiova1@uniba.sk; 4 Institute of Pathological Anatomy, Faculty of Medicine, Comenius University in Bratislava, Sasinkova 4, 811 08 Bratislava, Slovakia; pavel.babal@fmed.uniba.sk

**Keywords:** Hamperl cells, uterine NK cells, endometrium, histology, immunohistochemistry, medical education, Terminologia Histologica

## Abstract

Reproductive immunology is at the forefront of research interests, aiming to better understand the mechanisms of immune regulation during gestation. The relationship between the immune system and the implanting embryo is profound because the embryo is semi-allogenic but not targeted by the maternal immune system, as expected in graft-versus-host reactions. The most prominent cell population at the maternal–fetal interface is the population of uterine natural killer (uNK) cells. Uterine NK cells are two-faced immunologically active cells, bearing comparison with Janus, the ancient Roman god of beginnings and endings. Their first face can be seen as natural killer cells, namely lymphocytes, which are critical for host defense against viruses and tumors. Even though uNK cells contain cytolytic molecules, their cytotoxic effect is not applied to classical target cells in vivo, playing a permissive rather than a defensive role. Their second face is crucial in maintaining physiological gestation—uNK cells show critical immunomodulatory functions with the potential to control embryo implantation and trophoblast invasion, regulate placental vascular remodeling, and promote embryonic/fetal growth. Therefore, we believe that their current designation “natural killer cells” (the first “cytotoxic” Janus’s face) is misleading and inappropriate, considering their principal function is supporting and maintaining pregnancy. In this narrative review, we will focus on three lesser-known areas of knowledge about uNK cells. First, from the point of view of histology, we will comprehensively map the history of the discovery of these cells, as well as the current histological possibilities of their identification within the endometrium. To be brief, the discovery of uNK cells is generally attributed to Herwig Hamperl, one of the most influential and prominent representatives of German pathology in the 20th century, and his co-worker, Gisela Hellweg. Secondly, we will discuss the interesting aspect of terminology, since uNK cells are probably one of the human cells with the highest number of synonymous names, leading to significant discrepancies in their descriptions in scientific literature. From the first description of this cell type, they were referred to as endometrial granulocytes, granular endometrial stromal cells, or large granular lymphocytes until the end of the 1980s and the beginning of the 1990s of the last century, when the first publications appeared where the name “uterine NK cells” was used. The third area of present review is medical teaching of histology and clinical embryology. We can confirm that uNK cells are, in most textbooks, overlooked and almost forgotten cells despite their enormous importance. In the present narrative review, we summarize the lesser-known historical and terminological facts about uNK cells. We can state that within the textbooks of histology and embryology, this important cell population is still “overlooked and neglected” and is not given the same importance as in fields of clinical research and clinical practice.

## 1. Introduction

In recent years, views on the importance and functioning of the immune system during blastocyst implantation, placentation, and subsequent pregnancy have changed significantly. In the past, it was assumed that the mother’s immune system only plays a role in the tolerance of the semi-allogeneic embryo, which also contains paternal antigens. However, today’s opinions confirm that a woman’s immune system also plays a role in numerous other physiological changes during pregnancy, starting with implantation, through the formation of the decidua and placenta, and ending with the embryo’s and fetus’s growth. In addition, a woman’s endometrium, including immunologically active endometrial cells, plays an active role in all these events and not just a passive one, as previously thought [1].

The interaction between the embryo and the maternal immune system is dynamic and evolving. The endometrial stroma contains four main types of immunologically active cells: macrophages, T-lymphocytes, antigen-presenting dendritic cells, and uterine natural killer (uNK) cells. In contrast to other mucosal tissues, scattered lymphoid aggregates of B-lymphocyte and plasma cell infiltration are scarcely presented in healthy endometrial tissue [2]. Other cells of the immune system, such as eosinophils or mast cells, are present only in smaller numbers. In the non-pregnant uterus, uNK cells represent 26% of the endometrial immune cells during the late proliferation phase and 83% during the mid-secretory phase. uNK cell fluctuations during the menstrual cycle may reflect hormonal regulation of maternal immunity, thereby promoting tolerance at implantation [3]. During early pregnancy, uNK cells are the most abundant cell type at the maternal–fetal interface in humans, reaching their peak (making up 70% of the total lymphocytes in the endometrium) in the third month of pregnancy before undergoing a decline [4].

Bearing comparison with Janus, the ancient Roman god of beginnings and endings, uNK cells are two-faced immunologically active cells. Their first face can be seen as natural killer cells, namely innate lymphocytes, which are critical for host defense against viruses and tumors. Even though uNK cells contain cytolytic molecules, their cytotoxic effect is not applied to classical target cells in vivo, playing a permissive rather than a defensive role [5]. However, when exogenously stimulated in vitro, uNK cells can kill cellular targets, including semi-allogeneic cytotrophoblast cells [6]. Indeed, uNK cells can destroy cytomegalovirus-infected decidual stromal cells and are required for effective responses to *Chlamydia trachomatis* endometrial infection [7,8]. Their second face is crucial in maintaining physiological gestation; uNK cells show critical immunomodulatory functions with the potential to control embryo implantation and trophoblast invasion, regulate placental vascular remodeling, and promote embryonic and fetal growth. The uNK cells regulate vascular remodeling via the secretion of angiogenesis regulatory molecules, cytokines, and chemokines [9]. The consensus is that uNK cells are beneficial in early pregnancy. Additionally, uNK cells can retain a memory of pregnancy, suggesting that uNK cells remember encounters with previous placentae, perhaps explaining the longstanding observation that second and subsequent pregnancies are at lower risk of pre-eclampsia or miscarriage unless there is a change of partner, in which case the risk returns to that seen in first pregnancies [4,10,11].

As the bottom line, the first cytotoxic Janus’s face of uNK cells is suppressed significantly (perhaps even completely in vivo) during gestation. Therefore, we believe that their current designation of “killer” is misleading and inappropriate, considering their principal function is supporting and maintaining a pregnancy.

The uNK cells in the endometrial tissue are a unique cell population with divergent characteristics compared with peripheral blood NK cells. They differ in phenotype, probably origin, and function. Although we do not know the exact significance of uNK cells in the process of implantation and placentation, just as we do not know the significance of the involvement of these cells in the pathophysiology of recurrent implantation failure, in this review, we will focus on three lesser-known interesting facts about uNK cells. First, from the point of view of histology, we will comprehensively map the history of the discovery of these cells, as well as the current possibilities of their histological identification within the endometrium. Secondly, we will discuss the interesting aspect of terminology, since uNK cells are probably one of the human cells with the highest number of synonymous names, leading to significant discrepancies in their scholarly research descriptions. The third area of our interest is the medical teaching of histology and clinical embryology, within which uNK cells are among the most overlooked and almost forgotten cells despite their enormous importance.

## 2. Historical Overview of the Uterine Natural Killer (uNK) Cell’s Discovery

The discovery of uNK cells is generally attributed to Herwig Hamperl (1899–1976), one of the most influential and prominent representatives of German pathology in the 20th century [12]. Hamperl is one of the pioneers of fluorescence microscopy, and together with Max Haitinger, they performed the first systematic fluorescence staining in histology [13]. Hamperl (1950), in his first comprehensive work, refers to the newly discovered cells in the endometrium as “fluorescierende Körnchenzellen”, which can be translated as fluorescent granular cells or, in shortened form, as “fluorocytes” [14]. In his work, Hamperl mentions that similar cells—argentaffin (take up silver stain) macrophages named “Körnchenzellen”—were described in the inaugural dissertation of W. Dyx in 1941, but the work could not be accessed. In subsequent years, it was discovered that acidophil granulated cells located in the human endometrium and decidua had been observed many years ago by Marchand in 1904 [15] and Weill in 1921 [16], as mentioned by Hellweg in 1959 [17]. Hamperl initially preferred the name “F cells” or fluorocytes, due to the typical fluorescence of these granules in the frozen or paraffin tissue sections when exposed to UV light. They were described as cells 20–30 µm in diameter, with one round-to-oval nucleus and many small granules in the cytoplasm. These granules were described as a little bit larger than granules of eosinophils and usually densely packed in occurrence. In formalin-fixed sections, these cells were without color or were light yellow. They were found in the uterus, endometrial cysts, uterine tubes, breast tissue, cervix, and many locations. Hamperl [14] concluded that the appearance of these cells was typical for tissues with regular bleeding and supposed that these cells were ingesting hemoglobin and other degradation products of cells, using them for their function. In his work in 1954, Hamperl named these cells “endometriale Granulocyten” (i.e., endometrial granulocytes) [18]. Under Professor Hamperl, as the Head of the Institute of Pathology at the University of Bonn in Germany, Gisela Hellweg continued describing these cells in 1956. She called them “Endometrial Körnchenzellen—KZ cells”. Hellweg proposed features that distinguished “KZ cells” from neutrophils and eosinophils, such as different morphology of their nuclei and missing positivity for oxidase reactions. She also described their difference with plasma cells because of the atypical nucleus and missing staining affinity for methyl green-pyronin stain or picric acid. In contrast to mast cells, “KZ cells” contained fewer granules and were missing metachromasia, basophilia, and staining with aldehyde fuchsin. According to Hellweg, distinguishing them from lymphocytes was a much more complex issue. Nevertheless, she observed different arrangements of chromatin in the nucleus. Another distinct feature was that “KZ cells” granules lacked affinity to the May–Grünwald–Giemsa stain. Another important observation was the higher occurrence of these cells during the secretory phase of the menstrual cycle. Furthermore, she described that the granules of these cells were filled with protein substances. In contrast to the first description by Professor Hamperl in 1950, she described only those in the uterus [19].

Hamperl and Hellweg (1958) described these cells: “*In the endometrium, during the secretory phase and in the decidua up until three months’ gestation, cells appear which contain non-metachromatic granules. These are called granular endometrial stroma cells (Körnchenzellen or K cells). If the presence of K cells is followed during the normal menstrual cycle, it becomes evident that they are absent in the proliferative phase*”. The cited authors supposed that they originate from the undifferentiated stromal cell of the endometrium, just like decidual cells. They relied on the observation that they are located in the same places within the pars compacta of the endometrium and around the blood vessels. In addition, the authors added that “*The K cells were demonstrable in those regions where decidual cell formation had also occurred; that is, in islets of pseudodecidua in the ovary, or under the peritoneum, in a pseudodecidual reaction in the mucosa of the uterine tube with ectopic pregnancy, and endometriosis*” [20].

In the 1980s and early 1990s, after the routine introduction of immunohistochemistry and flow cytometry methods into practice, these uterine cells began to be referred to as “large granular lymphocytes”. At the same time, researchers began to investigate the phenotypic similarities and differences between them, peripheral lymphocytes, and NK cells [21,22,23,24,25]. Around 1990, they were finally identified by immunohistochemistry and flow cytometry as a type of natural killer cell with a distinctive phenotype, CD56^bright^, but lacking the other NK cell markers used at the time: CD16 and CD57 [11]. In that time, the first scientific publications that named this cell population as the currently known “uterine/decidual NK cells” were published [26,27,28].

## 3. Terminological Confusions around uNK Cells

There are probably few cells in the human body with as many different names as uNK cells. Eponymously, they are called Hamperl cells after their discoverer [29]. However, according to Winkelmann [30], many anatomical eponyms—including Hamperl cells—are only used by anatomists and have historical value at best. Therefore, they should be dropped from the medical curriculum and everyday clinical practice. Hamperl himself named them “K cells”. Surprisingly, this name also appears in contemporary textbooks focused on uterine pathology, such as in [31]. Another historical term, but at the same time an utterly misleading term in our opinion, is “endometrial stromal granulocyte”, which nevertheless appears in two contemporary histology textbooks [32,33]. The misleading nature of this term lies in the fact that uNK cells have a different origin, morphology, and function than granulocytes (white blood cells originating from the myeloid lineage). The officially valid and internationally accepted histological nomenclature “Terminologia Histologica” [34] refers to these cells in Latin as “cellula granularis endometrii”, with acceptable English equivalents: endometrial granular cells or endometrial natural killer cells (unlike the commonly known term uNK cells). To complicate the matter even further, the world-famous textbook of embryology by Moore et al. [35] uses the terms uNK cells and decidual NK (dNK) cells interchangeably. Moreover, some authors strictly discriminate uNK cells into endometrial NK cells and decidual NK (dNK) cells. Male et al. [36] argued that a specific repertoire of killer immunoglobin-like receptor (KIR) expression which reacts with the fetal HLA-C necessary for trophoblast invasion is found specifically in dNK cells. This makes them distinct from the endometrial NK cells found in the endometrium regardless of pregnancy. A similar strict distinction was discussed by Xie et al. [37], who subdivided uNK cells into non-pregnant endometrial NK cells, which renew over the course of the menstrual cycle. During the menstrual phase, they are discharged with the menstrual blood, becoming “menstrual blood NK cells”. On the other hand, dNK cells are pregnancy-associated NK cells which share some phenotypic similarities with endometrial NK cells but are nevertheless different.

Which cell name should we use when preparing scholarly research? The right way would be to use the term found in the Terminologia Histologica [34] (i.e., “endometrial granular cell”). However, if we search for this term in common databases such as Medline and PubMed, Google Scholar, or Web of Science, we will find that it is not used in the scientific literature (Table 1). In addition, as already mentioned, the term “granular cell” evokes a similarity with granulocytes, a cell population entirely dissimilar from NK cells from the developmental, functional, and morphological perspectives. Likewise, the alternative name according to Terminologia Histologica, which is “endometrial NK cell”, is used relatively rarely compared with other names. At the same time, we consider this name the most correct histologically, as these NK cells are located predominantly in the endometrium and not in the entire thickness of the uterine wall (which also includes the myometrium and perimetrium). However, we believe that most of the time, the official terminology should be adapted to everyday practice and not the other way: introducing artificially created new names into common practice, which would be unrealistic. Therefore, we recommend using the term “uterine NK cell” (i.e., “lymphocytus K uteri” in Latin) or, in the case of pregnancy and decidual transformation of the endometrium, the alternative term “decidual NK cell” (i.e., “lymphocytus K decidui”). These suggestions should be considered when preparing the second and updated edition of the Terminologia Histologica. For the sake of completeness, it is necessary to add that uNK cells can also be identified by the less specific name “tissue-resident NK cells of the uterus”. However, in addition to the population of uNK cells, other subpopulations of innate lymphoid cells are present in the endometrium. The phenotype and functions of other uterine innate lymphoid cells were, until now, poorly defined [38].

## 4. Current Histological Knowledge and Immunohistochemistry of uNK Cells

The uNK cells are sporadically present in the endometrium during the proliferative and early secretory menstrual phases. Their count rises substantially from the mid-secretory phase of the cycle. Their count reaches the maximum in the first trimester. Afterward, their number diminishes. At term of birth, there is only a minimal number of them present. They are also present in the endometrial glands and within the decidua basalis and parietalis, typically surrounding spiral arteries [39]. Studies also indicated that uNK cells are proliferative, especially in the secretory phase of the menstrual cycle, as they were positive for the proliferation marker Ki67 [40].

Morphologically, uNK cells correspond to large granular lymphocytes and belong to innate immunity. They represent 70% of maternal leukocytes during pregnancy. Typical characterization is through phloxinophilic cytoplasmic granules that stain darkly with periodic acid—Schiff staining (PAS reaction), indicating the presence of glycoproteins. These granules usually appear regular, growing in size and number until approximately two weeks of gestation. The granules differ between species in size and content. Human uNK cell granules contain cytotoxic mediators, namely perforin and granzyme. Even though uNK cells are not typically cytotoxic, after exposure to some protein (e.g., interleukin-2), they may become destructive and target extravillous trophoblast [41]. In all species, they have numerous organelles, including mitochondria, a well-developed Golgi apparatus, free ribosomes, and a rough endoplasmic reticulum [42]. The granules of uNK cells are larger compared with granules of peripheral NK cells. NK cells with larger granules are better cytokine producers [43]. Except for granules, uNK cells contain small oval and indented hyperchromatic nuclei [40], and uNK cells produce many cytokines and chemokines like GM-CSF, CSF1, CCL2, CCL3, CCL4, and XCL1 [44]. For the sake of completeness, it is necessary to mention that tissue-specific NK cells are found not only in the uterus but also in various other organs and tissues of the human body (e.g., thymus, spleen, liver, or adipose tissue). All these subpopulations of NK cells may have differences not only in anatomical location but also in transcription factor requirements, cytokine receptor dependence, and functions [45,46].

Historically, uNK cells were characterized as lymphoid cells that were positive for the common leucocyte antigen (CD45), T-cell antigen CD2 (E-rosette receptor), CD7, CD38 (OKT 10), CD45RO (UCHL1), and MT1-MMP. However, uNK cells are negative for classic natural killer cell markers like Leu 7 and Leu 11 (CD16) [47,48,49,50]. Nowadays, uNK cells are typically defined by their unusual phenotype, which is different from that of peripheral blood cells. Unfortunately, few studies have complexly characterized the phenotype of uNK cells. On the one hand, uNK cells share a similar expression profile of CD56, CD57, CD94, and CD16 with peripheral blood CD56^bright^ NK cells. On the other hand, uNK cells share a similar expression profile of KIR receptors CD158b and NKB1 with CD56^dim^ NK cells, and they also lack the expression of l-selectin. Furthermore, uNK cells were shown to express the activation markers HLA-DR and CD69 [51]. Additionally, it has long been appreciated that uNK cells do not form a uniform population. As in peripheral NK cells, there is cell-to-cell variation in the precise combination of NK cell receptors that are expressed. Recently, however, new single-cell RNA sequencing techniques have enabled an unbiased approach to these cells, and three major subpopulations were identified in first-trimester decidua, originally called dNK1, dNK2, and dNK3 cells [52]. While the function of each subset is unknown, dNK1 cells express transcripts that suggest a role in extravillous trophoblast recognition and interaction; dNK2 cells potentially have anti-inflammatory functions; and dNK3 cells could play a role in extravillous trophoblast regulation. The phenotypes of these different subpopulations of uterine and decidual NK cells were recently reviewed by Male and Moffett [11].

Several research papers used various morphological approaches to study uNK cells, like classic histology, ultrastructural analysis, or immunohistochemistry. Morphological studies elucidated that uNK cells undergo profound changes in the decidua. In mice, uNK cells were observed to form membrane-bound granules, quickly and dramatically increasing in size up to 80 μm [53]. Kusakabe et al. [54] performed a study which examined the morphological changes in uNK cells undergoing cell death during different stages of gestation. The main observations were not surprising, as the uNK cells displayed nucleus condensation, size reduction, and changes in the structure of their granules.

The recent principal histopathological approach in the study of uNK cells in uterine bioptic samples is immunohistochemistry. Most published immunohistochemical studies evaluated the CD56-positive cells in the endometrium and decidua of patients with unexplained recurrent implantation failure and habitual abortion as absolute numbers of CD56-positive cells per square millimeter [55,56,57,58,59,60]. The advantage of such a histological approach is the possibility of studying the cyto-architectonics in the endometrial tissue. Histological specimens allow us to study the mutual spatial relationships between different cells of the endometrium. The disadvantage of the histopathological approach is that uNK cell counting is mostly semi-quantitative, which depends on both the quality and representativeness of the biopsy sample, as well as on the experience of the examining physician (pathologist). The second laboratory approach—which is of significant importance in everyday clinical practice—is the endometrial immune phenotyping of biopsy samples or uterine lavage fluid (and eventually experimentation with menstrual blood) in women with unexplained recurrent implantation failure through flow cytometry [61,62,63,64,65]. More recently, molecular biological approaches (e.g., using single-cell RNA sequencing or mass spectrometry-based proteomics) may also be suitable for making detailed molecular and cellular maps of endometrium in health and disease [66]. These emerging molecular biological technologies hold great promise for providing novel insights into the molecular mechanisms underlying endometrial receptivity and the role of uterine NK cells during successful or unsuccessful embryo implantation [67].

## 5. Functional Overview of uNK Cells during Implantation

NK cells play an important role in our immune system. In contrast to mice, human implantation is not dependent on an estradiol surge. Also, decidualization is not reliant on implanting an embryo but through elevated progesterone levels and intracellular cAMP. Human uNK and murine uNK cells accumulate in the endometrium, similarly helping vascular adaptation and trophoblast invasion. During implantation, uNK cells are the most common immune cells in the uterus and at the maternal–fetal interface. The most crucial step for implantation is tolerating a semi-allogenic embryo by avoiding cytotoxic activity during the implantation window. Peter Brian Medawar first proposed this concept of “fetal allograft” in the 1950s. Three fetal factors were described considering this issue: anatomical separation of mother and fetus, a decreased antigenic property of the fetus, and immunological inertness of the maternal immune system. This proposal influenced many researchers. Although uNK cells are supposed to have minimal cytotoxicity, they can become cytotoxic mainly during the proliferative phase of the menstrual cycle, preventing microbial infection [68]. As the menstrual cycle proceeds, the cytotoxic function of uNK cells becomes weaker, and their count increases. This process is probably activated by sex hormones, mainly through the influence of progesterone [69]. Some authors suggest that if fertilization does not occur, then they will undergo apoptosis before the next cycle as the progesterone level decreases [70].

**Figure 1:**
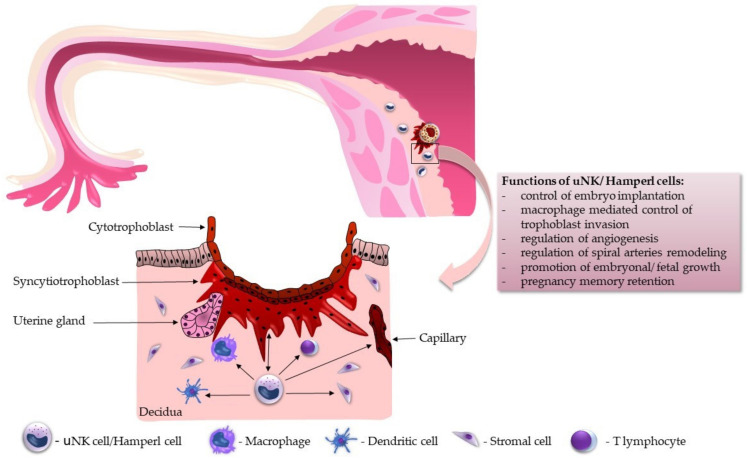
uNK cells are vital for successful embryo implantation, placentation, and pregnancy promotion.

Understanding the immune mechanisms during implantation is essential. The optimally balanced immune response at the maternal–fetal interface plays a deciding role in the semi-allogeneic embryo’s endometrial receptiveness during the implantation window. In the mid-luteal phase, nearly all immune cells from our adaptive immunity leave the endometrium. At the same time, innate immune cells (e.g., uterine leukocytes (macrophages, especially embryonic or fetal macrophages called Hofbauer cells, as well as uNK cells)) invade the endometrium and dramatically increase in number, representing at least 15% of all cells in the decidua [70,71]. The adaptive immune system is controlled mainly by regulatory T cells (Tregs), a subgroup of suppressor CD4+ T cells. They secure immune tolerance, coordinate inflammation, and support vascular adaptation [68]. The innate immune system is impacted by the Th-1 and Th-2 cytokine balance. Differentiation of local immune cells in the beneficial or deleterious pattern depends on Th-1 or Th-2 predominance. In a Th-1-dominant environment, macrophages differentiate into deleterious M-1 macrophages, uNK cells differentiate into lymphokine-activated killer cells, dendritic cells differentiate into deleterious DC-1, and T cells differentiate into harmful Th-17 cells. All these cells become able to target and kill the embryo. On the contrary, in a Th-2-dominant environment, macrophages differentiate into M-2 macrophages for settling adhesion, uNK cells become angiogenic and immunotropic, dendritic cells differentiate into DC-2, providing effective communication, and T cells differentiate into Tregs to promote local tolerance [72]. For successful implantation, the necessary spiral artery remodeling is initiated by uNK cells, where uNK cells surround spiral arteries and produce angiogenic growth factors like vascular endothelial growth factor (VEGF) or stromal cell-derived factor (SDF). These released factors can enhance or inhibit the invasion, highlighting the importance of uNK cells in supporting successful pregnancies [38,73]. In addition, uNK cells probably increase the vascular smooth muscle reorganization necessary for spiral artery remodeling [74]. Moreover, by producing killer cell immunoglobulin-like receptors (KIRs), uNK cells recognize fetal extravillous trophoblast and its human leukocyte antigen (HLA). The interaction between KIRs and the HLA of the embryo influences the secretion of angiogenic factors by uNK cells. There is a significant polymorphism between both HLA and KIR genes. Combinations of specific haplotypes can lead to disorders like recurrent pregnancy loss, pre-eclampsia, preterm labor, fetal growth restriction, or even birth due to defect placentation. The main functional aspects of uNK cells are depicted in Figure 1.

## 6. Current Knowledge of uNK Cells in Histology and Embryology Textbooks

The present paper clearly shows how essential uNK cells are in early embryogenesis and placental development. Nevertheless, we are surprised that even after more than 70 years since their detailed histological description, there is no mention of uNK cells in most of the latest textbooks on histology and embryology used worldwide [75,76,77,78,79,80]. In the 6th Edition of *Human Embryology and Developmental Biology*, the issue of tolerance of the mother’s immune system toward the semi-allogenic embryo is discussed only in a few sentences, with a conclusion that “*a real understanding of how this is accomplished has resisted years of intensive research*” [81]. On the other hand, *Larsen’s Human Embryology* discusses the problem of immune tolerance in more detail. However, uNK cells are nowhere to be mentioned. Only the role of Tregs is recognized [82]. Brief information about uNK cells (often under different synonymous designations) can be found only in four internationally used histology textbooks and one embryology textbook. Table 2 summarizes these findings.

## 7. Conclusions and Future Perspectives

In the last few years, there has been significant progress in understanding implantation and the immune processes connected to it and in understanding uNK cells. Questions regarding their origin and function are much clearer and may be used positively in many directions. As mentioned above, many gynecological and obstetrical complications seem to be connected to their alterations. Around 20–30% of women with idiopathic recurrent miscarriages or recurrent implantation failure show, according to some studies, altered uNK cell counts [57]. The observation of significantly increased uNK cell levels in the endometrium of women with recurrent implantation failure or recurrent miscarriages may point to an underlying disturbance of the immune milieu, culminating in implantation or placentation failure [85]. Available tests using immunohistochemistry and flow cytometry enabled us to find out more about uNK cell counts for different patients with different diagnoses. A better understanding of uNK cell functions will be essential in identifying the mechanisms promoting implantations and possibly providing better pregnancy outcomes. Although the uNK cell research is still far from translation to routine clinical practice, several possible future treatment avenues can successfully manage pregnancy-related conditions, including recurrent implantation failure and recurrent miscarriages. The first possible approach is controlling the negative hormone impact on uNK cell function, which affects decidualization, placentation, and pregnancy success. Kanter et al. [86] experimentally demonstrated that uNK cells are very receptive toward hormonal signals. The authors conducted a prospective cohort study and found that hormonal stimulation associated with in vitro fertilization (IVF) techniques affects uNK cell distribution deleteriously, hindering their ability to promote trophoblast invasion. Although the textbook *Infertility in Practice* mentions that there is no indication that immune therapy might work and provide an optimal risk-to-benefit ratio [87], there is a speculative yet innovative therapeutic approach: the use of platelet-rich plasma (PRP). Benkhalifa et al. [88] summarized that immunomodulation through PRP could positively influence the risk of implantation failure and pregnancy loss in IVF patients. Ban et al. [89] also reported that the infusion of leucocyte-poor PRP is an effective tool for managing recurrent implantation failure. We hypothesize that PRP’s direct intrauterine application might work through the positive influence on uNK cells, promoting their normal function. Other experimental forms of therapy directed on uNK cells have been tried with different levels of success, and controversies exist regarding the benefits of intravenous intralipid therapy [90], recombinant granulocyte colony-stimulating factor injection [91,92], steroids (10 mg of prednisone once daily from the day of starting endometrial preparation) [93], or possible mesenchymal stem cell application in the endometrium [94].

Unfortunately, as summarized in a recent paper by Sfakianoudis et al. [95], the exact pathophysiological mechanisms of how uNK cells may contribute to recurrent implantation failure or recurrent miscarriages are still obscure and elusive. Some researchers indicated that a higher count of uNK cells is associated with the risk of recurrent miscarriages [96], while others found no association and reported a failure of uNK quantitative evaluation to predict pregnancy outcomes [97]. The lack of knowledge on the topic is reflected in the conclusion of the systematic review and meta-analysis by Seshadri and Sunkara [98], who suggested that uNK cell evaluation and immune therapy should be implemented only in the setting of clinical research. On the other hand, a meta-analysis published by Von Woon et al. [85] observed a significant increase in uNK cell count, implying that immune homeostasis is crucial in normal implantation and placentation. The authors highlighted the lack of a standardized protocol for a routine clinical evaluation of the uNK cell count and activity, creating a hurdle which has prevented the straightforward bench-to-bedside clinical utility of such evaluations thus far. The most probable explanation for the ambiguous results is that an increased or decreased count of uNK cells can be detrimental. The most crucial factor is their functional integrity. Normally functioning but quantitatively diminished uNK cells can be as harmful as a normal amount of inadequately functioning uNK cells or a high amount of functionally compromised and overreactive uNK cells.

All in all, uNK cell research needs to address several issues before it can be implemented clinically, namely the standardization of uNK cell evaluation, more profound insights into how uNK cells contribute to immune homeostasis and immune suppression and how exactly they interact with all the components of the decidua during pregnancy, and finally yet importantly, the development of a precise methodology of uNK cell targeting within the state-of-the-art immune therapy of pregnancy-related conditions.

## Figures and Tables

**Table 1:** Terminology of uNK cells in various scientific databases.

	PubMed and Medline	Google Scholar	Web of Science
Uterine NK cells	182 results	5110 results	392 results
Decidual NK cells	182 results	6630 results	324 results
Endometrial granular cells	9 results	79 results	3 results
Endometrial NK cell	33 results	823 results	13 results

**Table 2:** Internationally recognized histology and embryology textbooks, which contain some basic information about uNK cells.

Title of Textbook	Authors	Description
*The Developing Human*. 11th edition. Year 2020, Page No. 108.	Moore K.L., Persaud T.V.N., Torchia M.G. [35]	In addition to averting T cells, extravillous trophoblast cells must also shield themselves from potential attack by NK lymphocytes. Maternal lymphocytes within the pregnancy-associated decidua include a high portion (65–70%) of NK cells and a low portion (10–12%) of T cells. Decidual or uterine NK cells are distinct from peripheral blood NK cells’ phenotype and function in having poor cytotoxicity for extravillous trophoblast cells.
*Wheater’s Functional Histology*. Sixth Edition. Year 2014, Page No. 356.	Young B., O’Dowd G., Woodford P. [32]	Secretory endometrium: Endometrial stromal granulocytes, which are probably large granular lymphocytes, are found in the stroma at this stage.
*Memorix Histology*. 1st edition. Year 2018, Page No. 397.	Balko J., Tonar Z., Varga I. [83]	Endometrial granular cells and Hamperl cells have a T lymphocyte of a spherical shape and markedly lobular nucleus. Present mainly in the secretory phase of the menstrual cycle. Realizing cytokines, which affect the growth and multiplication of vessels in the mucosa.
*Histology and Cell Biology*. Fourth Edition. Year 2016, Page No. 699.	Kierszenbaum A.L., Tres L.L. [84]	The decidual reaction involves the production of immunosuppressive substances (mainly prostaglandins) by decidual cells to inhibit the activation of natural killer cells at the implantation site.
*Histology for Pathologists*. Fifth edition. Year 2020, Page No. 1080.	Mills S.E. (Ed). [33]	A second prominent cellular constituent (after stromal cells), particularly in the late secretory phase and during pregnancy, is what historically has been referred to as the “stromal granulocyte” but is now known to be a uterine NK cell. These are rounded cells with bilobed nuclei and pale cytoplasm containing eosinophilic granules. Their immune profile differs from that of blood NK cells. Their number appears to be positively correlated with the degree of predecidualization or decidualization in the surrounding endometrium. Indeed, the number of such cells was used by Noyes as a dating criterion. This close association has suggested to some workers that uterine NK cells play a role in the control of trophoblast invasion and spiral artery remodeling and may be important in initiating and maintaining decidualization. Alternatively, the death of uterine NK cells might be an early event in the onset of endometrial breakdown at menstruation.

## Data Availability

No new data were created or analyzed in this study. Data sharing is not applicable to this article.
